# Case Report: Personalized treatment in an advanced lung adenocarcinoma patient with EGFR exon 21 L858R mutations based on the patient-derived tumor organoids

**DOI:** 10.3389/fonc.2026.1742943

**Published:** 2026-05-12

**Authors:** Fan Zhang, Yuxiang Wang, Xi Zhang, Yuting Bai, Li Zhang, Shuqing Wei

**Affiliations:** 1 Department of Comprehensive Medicine, Shanxi Province Cancer Hospital/Shanxi Hospital Affiliated to Cancer Hospital, Chinese Academy of Medical Sciences/Cancer Hospital Affiliated to Shanxi Medical University, Taiyuan, China; 2 Ultrasonic Department, Shanxi Province Cancer Hospital/Shanxi Hospital Affiliated to Cancer Hospital, Chinese Academy of Medical Sciences/Cancer Hospital Affiliated to Shanxi Medical University, Taiyuan, China

**Keywords:** advanced lung adenocarcinoma, EGFR mutations, patient-derived tumor organoids, personalized treatment, sacituzumab govitecan

## Abstract

Lung cancer is the most frequently diagnosed cancer and the leading cause of cancer deaths worldwide. Despite substantial therapeutic advances, the prognosis of lung cancer patients remains dismal. Here, we report a case of advanced lung adenocarcinoma harboring EGFR exon 21 L858R mutations who finally achieved partial response (PR) in the lung lesion following patient-derived tumor organoid (PDTO)-guided personalized therapy, even after multiple lines of systemic treatment. In May 2021, a 52-year-old woman was diagnosed with right lung adenocarcinoma (cT3N2M1c IVB), accompanied by multiple metastases. Although Osimertinib was initiated as first-line therapy based on the identified EGFR exon 21 L858R mutations, the patient experienced disease progression. Subsequent treatment with ivonescimab plus pemetrexed disodium was administered, but disease progression persisted. Based on serial organoid drug sensitivity testing, repeated adjustments to the therapeutic regimen were made, and the patient ultimately achieved PR in the lung lesion. Our case demonstrates that the lung cancer organoids serve as a powerful preclinical platform for individualized treatment selection in patients with advanced lung adenocarcinoma via rapid functional drug screening. This personalized strategy shows great potential to optimize clinical outcomes for heavily pretreated patients.

## 1 Introduction

Lung cancer is the most frequently diagnosed cancer and the leading cause of cancer deaths worldwide, with approximately 2.5 million new cases and 1.8 million deaths reported in 2022 (1). Despite substantial advances in understanding tumor biology, optimizing treatment options and identifying predictive biomarkers over the past few decades, the prognosis of lung cancer patients remains dismal. Recently, molecular profiling has greatly promoted the development of genomics-based precision medicine for lung cancer by linking genetic alterations to targeted drugs. However, such gene-drug associations are often compromised by tumor heterogeneity (2). Lung cancer patients harboring identical genomic alterations may exhibit variable clinical responses even when treated with the same therapeutic regimen (3). Moreover, genotype-based analyses are generally insufficient to predict the patient response to chemotherapy (4). Therefore, the advancement of precision oncology urgently requires functionally relevant *in vitro* models that recapitulate the biological characteristics of the original tumor to accurately formulate personalized treatment strategies.

As three-dimensional (3D) *in vitro* culture systems, tumor organoids self-differentiated from cells with stem cell features are increasingly applied in personalized medicine and cancer research. They can simulate the biological, genetic and molecular characteristics while maintaining the intricate cellular heterogeneity and spatial architecture of the original tumor (5). Currently, the patient-derived tumor organoids (PDTOs) have been established successfully across multiple cancer types, including lung cancer, colorectal cancer, pancreatic cancer and breast cancer, and have emerged as a promising platform for studying tumor biology, developing personalized therapies and facilitating drug discovery (6–9). Through whole-exome sequencing and RNA sequencing, a detection rate of 92.7% in driver mutations from the primary tissue was observed in advanced lung cancer organoids (LCOs), and effective therapies for novel molecular targets were also identified using PDTOs (10). In a real-world study, LCOs not only recapitulated the pathological and genomic characteristics of the original tumor faithfully, but also predicted clinical treatment response accurately, unveiling the great potential of LCOs in guiding personalized treatment of lung cancer patients (11). Here, we report a case of advanced lung adenocarcinoma harboring EGFR exon 21 L858R mutations who achieved partial response (PR) in the lung lesion following PDTO-guided personalized therapy, despite failure of multiple prior lines of systemic treatment.

## 2 Case presentation

A 52-year-old woman was admitted to our hospital for further management, with the chief complaint of targeted therapy for the right lung adenocarcinoma for 21 days. She had a history of uterine fibroids, but denied a history of hypertension, diabetes mellitus, smoking, alcohol consumption, and any relevant family history of malignancy. In April 2021, a space-occupying lesion in the right lung was detected during a physical examination. In May 2021, she was diagnosed with right lung adenocarcinoma (cT3N2M1c IVB), accompanied by multiple distant metastases, including the lymph nodes, pleura, brain, liver, adrenal glands, and bone. Subsequent genetic profiling of the patient’s tumor tissue identified somatic EGFR exon 21 L858R mutations accompanied by EGFR copy number gain. Accordingly, Osimertinib (80 mg/d) was initiated as the first-line targeted therapy.

In December 2022, the patient experienced progressive disease (PD) according to Response Evaluation Criteria in Solid Tumors (RECIST) version 1.1, and Osimertinib was discontinued. In February 2023, she presented with left lower limb pain and received local tomotherapy, combining with 6 cycles of treatment with Pemetrexed disodium + Carboplatin + Bevacizumab. However, PD occurred again in August 2023.

In March 2024, the patient was treated with Furmonertinib combined with Anlotinib, but developed intolerable adverse reactions including nausea, blurred vision, and somnolence in May 2024. Subsequently, Ommaya reservoir placement and spinal subarachnoid peritoneal shunting were performed immediately, in combination with intrathecal injection of Pemetrexed Disodium. Meanwhile, the blood and malignant pleural effusion of the patient were respectively collected for ctDNA testing and organoid culture after obtaining the informed consent form. The patient was sampled at four different time points, and organoids from malignant pleural effusions were all successfully established. Briefly, the drainage bag containing malignant pleural effusions of >250 mL was transported to the laboratory [Kingbio Medical Co., Ltd., Chongqing, China] on ice, and centrifugation was performed to collect the tumor cells. Red cell lysing procedures were conducted, if necessary, which mainly depended on the sediment condition. Multi-well plates were used for seeding the tumor cells and Matrigel suspension when Matrigel was added and mixed. Once the droplets were solidified, appropriate amount of Jiabili^®^ organoid medium for lung cancer was supplemented. Subsequently, multi-well plates were placed in a 37 °C, 5%CO_2_ incubator for culture, with the culture medium replaced every 2–3 days. The organoids used in the testing were all at passage 1, and the time from sample collection to testing was 13, 19, 16 and 8 days, respectively. When organoids grew like solid spheroids with around 70-μm diameter, drug sensitivity testing was performed (12). The established organoids were first digested into single cells and resuspended in Jiabili^®^ tumor tissue basic medium II. After counting, the cell suspension was mixed with Matrigel and Jiabili^®^ lung cancer organoid medium, seeded into multi‐well plates, and incubated at 37 °C for 24 hours. Pre‐prepared drug solutions were then added for 72 hours of culture. Ultimately, the number of viable cells in the organoids was assessed using the CellTiter‐Glo^®^ Luminescent Cell Viability Assay. Specifically, luminescence signals were normalized to the vehicle control (defined as 100% viability), and the inhibition rate at each drug concentration was calculated accordingly. The concentration gradient for each drug was determined based on its clinically achievable blood concentration, typically from 100 μM to 0.1 nM. Dose-response curves were generated based on log-transformed drug concentrations, and half-maximal inhibitory concentration (IC_50_) values were estimated using a four-parameter logistic nonlinear regression model in GraphPad Prism. All conditions were tested in at least triplicate wells, and the results were presented as mean ± standard deviation. When 50% inhibition was not achieved within the tested concentration range, the IC_50_ value was recorded as greater than the highest tested concentration. Drug sensitivity was further classified into five categories according to the quintile distribution of inhibition rates observed under the tested conditions. All reagents used for organoid culture and drug sensitivity testing were provided by Kingbio Medical Co., Ltd., Chongqing, China.

**Figure 1:**
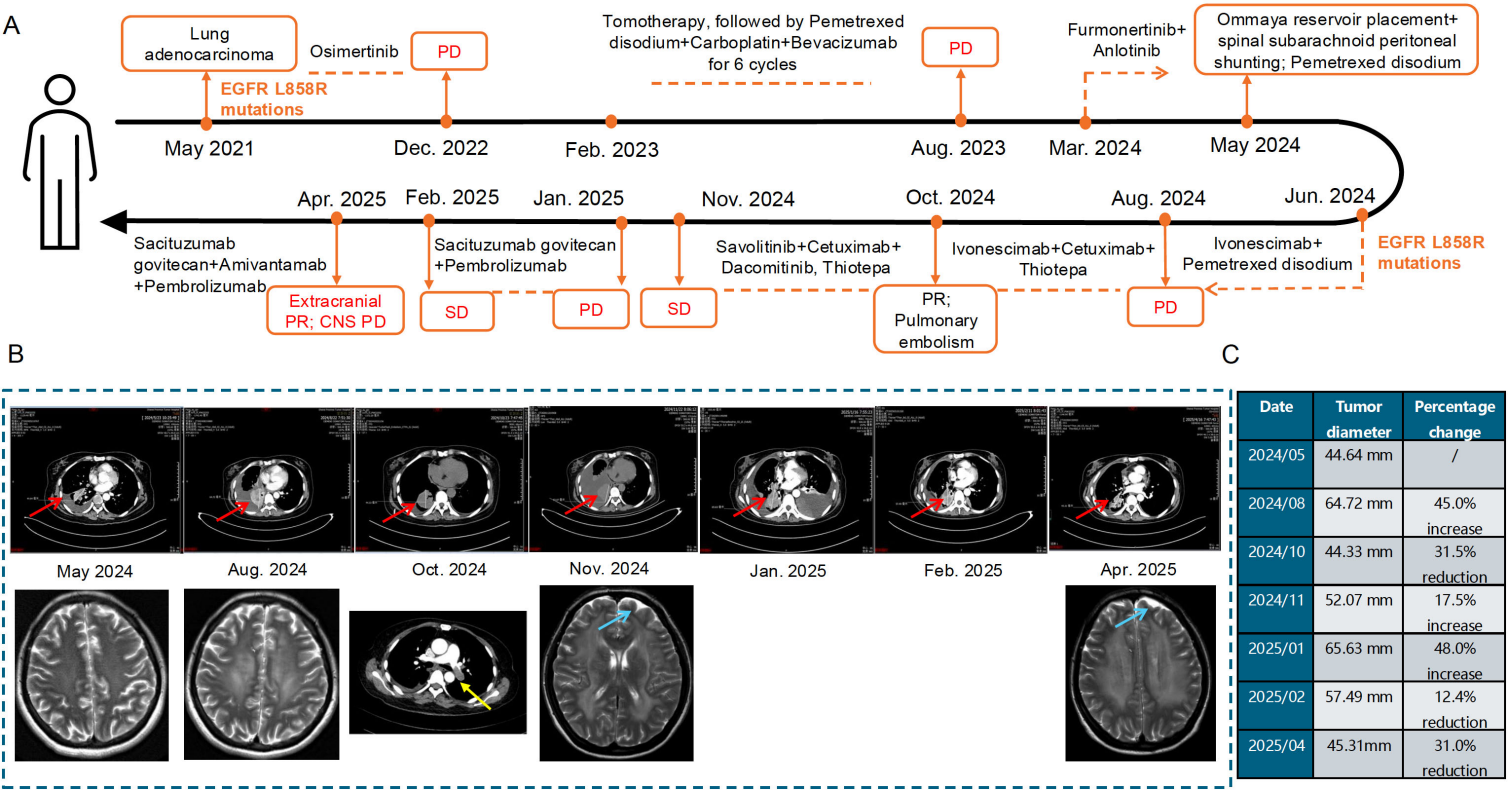
The diagram of diagnosis and treatment process **(A)**, the chest and brain CT images of the patient during the treatment **(B)**, and changes in lung tumor size during the treatment based on CT imaging **(C)**. Red, blue and yellow arrows head towards the lung lesion, the lesion in the central nervous system and pulmonary embolism, respectively.

**Figure 2:**
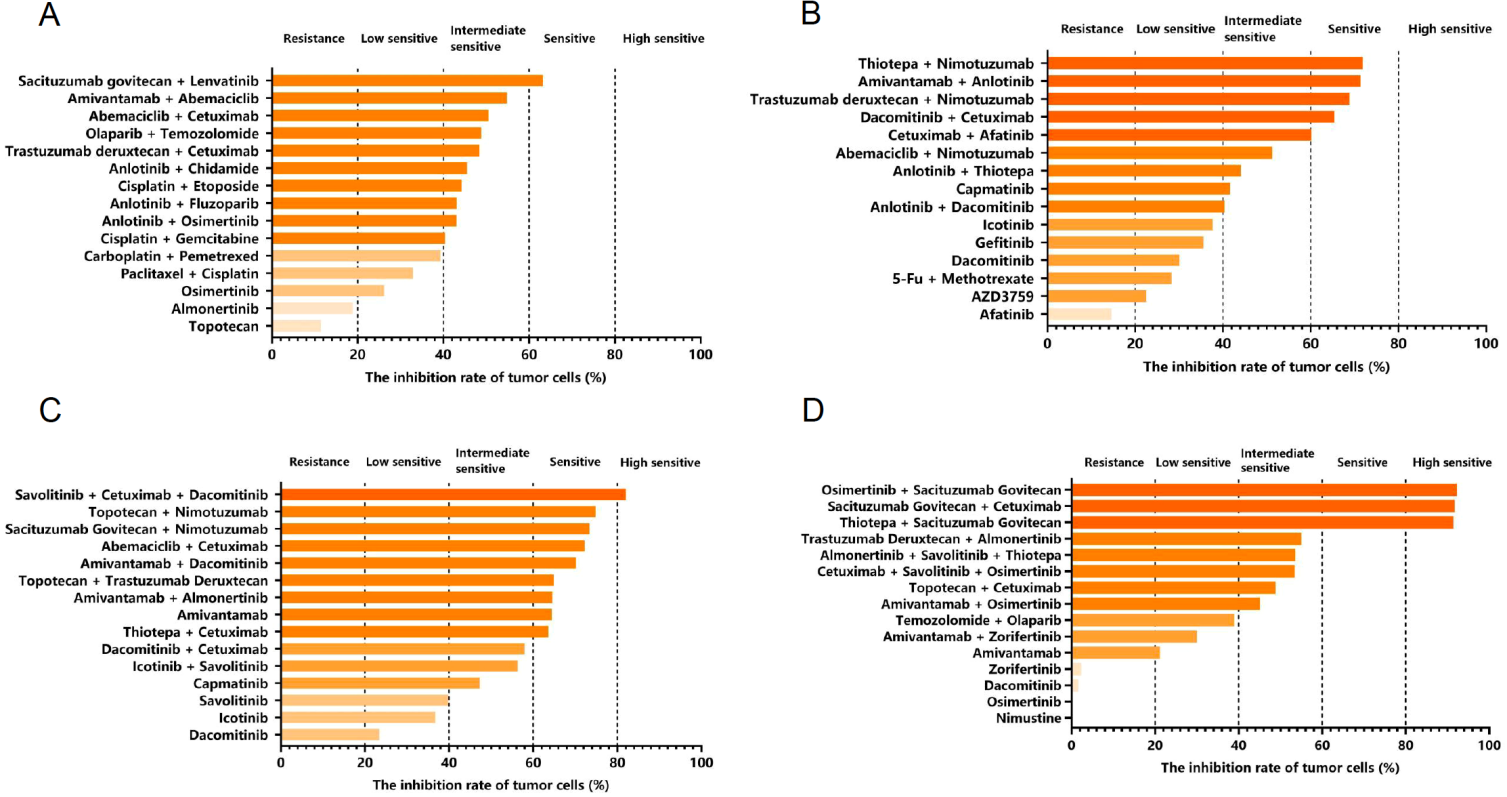
The multiple drug sensitivity testing results based on the organoids established from the malignant pleural effusion of the patient with advanced lung adenocarcinoma, including July 2024 **(A)**, September 2024 **(B)**, November 2024 **(C)** and February 2025 **(D)**. Drug sensitivity was classified into five categories according to the quintile distribution of inhibition rates, including high sensitive (>80%), sensitive (60%–80%), intermediate sensitive (40%–60%), low sensitive (20%–40%) and resistant (<20%).

**Figure 3:**
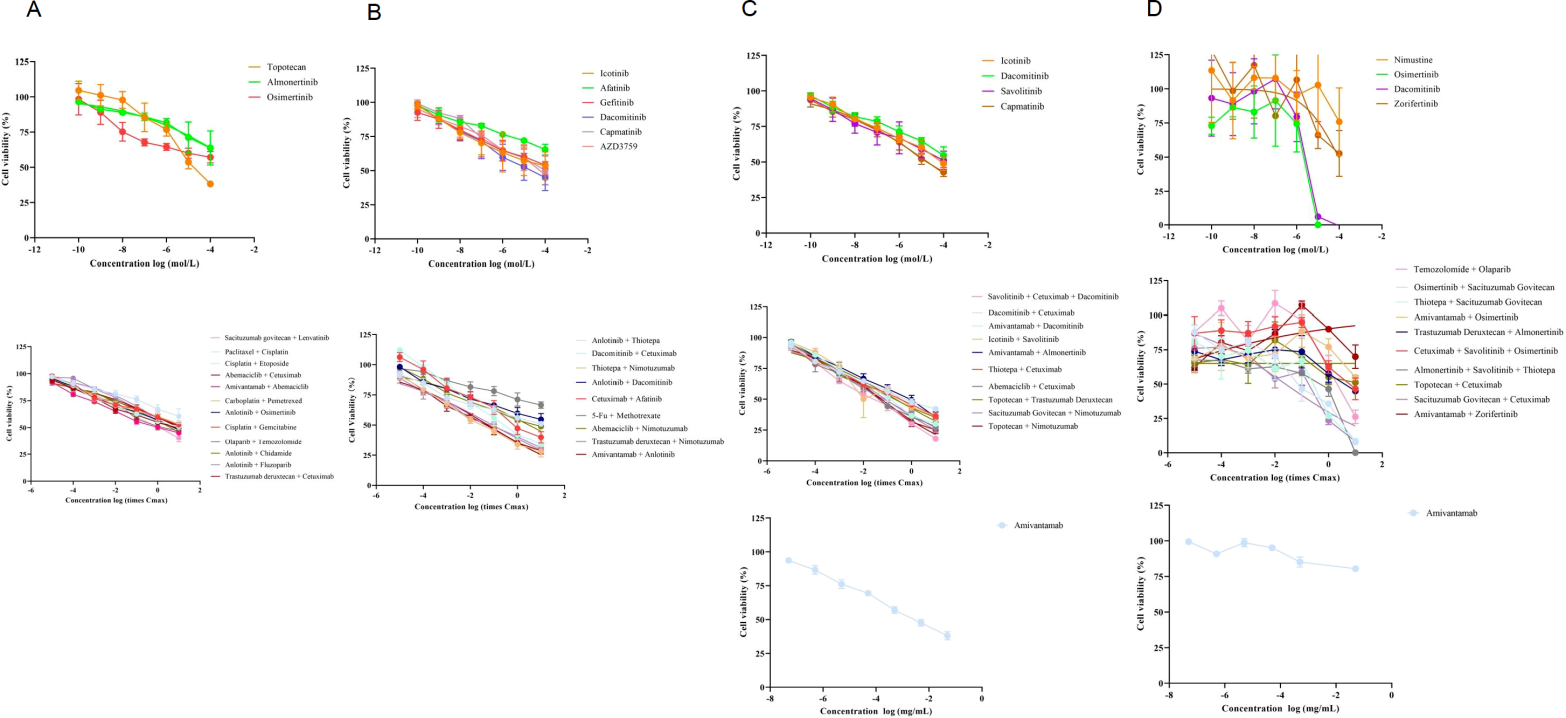
Dose-response curves of the advanced lung adenocarcinoma organoids treated with single agents and combined therapies at different time points, including July 2024 **(A)**, September 2024 **(B)**, November 2024 **(C)** and February 2025 **(D)**.

ctDNA testing further confirmed that the patient had EGFR exon 21 L858R mutations and EGFR amplification. Based on a recent phase III trial involving EGFR-mutant non-squamous non-small cell lung cancer patients irresponsive to EGFR-TKI therapy (13), the patient was given systemic treatment with Ivonescimab (800 mg, Q3W) and intrathecal injection of Pemetrexed Disodium (20 mg). In August 2024, the chest computed tomography (CT) examinations showed significant enlarged lesions in the right lung than before, and PD was assessed according to RECIST version 1.1 (**Figure 1**). In combination with the results of the first organoid drug sensitivity testing, the patient was treated with Ivonescimab (800 mg, Q3W), Cetuximab (600 mg, Q2W) plus intrathecal injection of Thiotepa (10 mg, Q1W) (**Figures 2A**, **3A**). On October 14, 2024, the lesions in the right lung were reduced significantly, but pulmonary embolism occurred (**Figure 1**), thus Ivonescimab was discontinued.

According to the second organoid drug sensitivity results, the patient was treated with Dacomitinib (30 mg Qd) and Cetuximab (600 mg Q2W), in combination with intrathecal injection of Thiotepa (10 mg QW) (**Figures 2B**, **3B**). Subsequently, it was informed that the patient was highly sensitive to Savolitinib, Cetuximab, and Dacomitinib based on the third organoid drug sensitivity results (**Figures 2C**, **3C**), thus she was treated with Savolitinib (400 mg, Qd), Cetuximab (600 mg, Q2W) and Dacomitinib (30 mg, Qd), and intrathecal injection of thiotepa was continued. In November 2024, the chest CT showed no obvious changes in the right lung lesions, but a lesion in the central nervous system (CNS) appeared through the brain CT (**Figure 1**). In January 2025, the lung lesions further enlarged (**Figure 1**).

The fourth organoid drug sensitivity testing was conducted immediately, and the results showed that the patient was highly sensitive to the treatment regimens with Sacituzumab govitecan (**Figures 2D**, **3D**). Therefore, Sacituzumab govitecan (200 mg, d1, d15) was used in combination with plus Pembrolizumab (100 mg, Q3W). On February 11, 2025, the right lung lesions shrank (**Figure 1**). In April 2025, the right lung lesions were decreased significantly than before, but the lesion in the CNS enlarged (**Figure 1**). The patient was treated with Sacituzumab govitecan, Amivantamab and Pembrolizumab. Currently, radiation therapy is undergoing.

## 3 Discussion

The formulation of treatment regimens is inaccurate only based on the findings of the genetic profiling and other biomarkers (14, 15). The molecular mechanisms underlying the response to therapies for most cases of lung cancer, especially for primary drug-resistant cases, are not entirely clear. Although next-generation sequencing holds promise for identifying targetable alterations, resistance to targeted therapy, such as tyrosine kinase inhibitors (TKI), usually appears because of secondary alterations or bypass signaling pathways (16). PDTOs, an innovative platform for drug testing and biomarker validation, play a crucial role in high-throughput drug screening. For lung cancer management, combination of LCOs may be a promising step toward driving precise medicine. In our study, the patient with advanced lung adenocarcinoma exhibited resistance to Osimertinib, an orally administered EGFR-TKI selectively targeting the activation of EGFR mutations, although EGFR exon 21 L858R mutations were detected. In combination of multiple PDTO-based drug sensitivity testing results, PR was finally achieved in the lung lesion, suggesting that PDTO-based drug sensitivity testing may serve as an effective predictive assay for tailoring the individual treatment regimens to improve the prognosis of the patients with advanced lung cancer at the individual level.

Unlike conventional 2D cancer cell lines, PDTOs can faithfully retain the cellular architecture, heterogeneity, and microenvironmental interactions of the original tumor, which are essential for the precise simulation of tumor biology (17). LCOs, a powerful platform for bridging basic and clinical research, enable researchers to study the disease in the previously unattainable methods by capturing the genetic, molecular, and microenvironmental characteristics of the original tumor (18). They have been confirmed to be a powerful platform for personalized treatment because the PDTOs originated from the individuals can be used to predict the response to specific drugs at the patient-specific level, thereby promoting the optimization of treatment regimens to improve the clinical outcomes (19). Moreover, PDTOs also exhibit a certain potential in reformation of drug development by offering more predictive data on the treatment response (20). Shi et al. found that the PDTOs from surgically resected tissues of non-small cell lung cancer retained the sensitivity of the matched parental tumor to targeted agents, contributing to identifying or validating biomarker-drug combinations (21). Liu et al. assessed the clinical benefit of adjuvant chemotherapeutic agents through establishment of early- and intermediate-stage LCOs and discovered that combination of non-platinum gemcitabine and vinblastine showed promising results regarding the suppression of cell proliferation and intra-organoid cell death (22). For advanced lung cancer, however, surgical samples are not easily available. In our study, the LCOs from malignant pleural effusions were successfully established multiple times, indicating that malignant pleural effusions may be an ideal source for LCO establishment. This is consistent with previous studies showing that patient-derived malignant pleural and peritoneal effusions are feasible and amenable to conducting *in vitro* drug testing in a personalized pattern (23).

Although next-generation sequencing exhibits good performance in detecting clonally dominant alterations in the context of modern precision medicine, but the drug resistance remains the greatest challenge for the targeted therapy. In our study, the patient presented EGFR-TKI-resistant disease, but did not show any resistant mutations. Therefore, the PDTO-based drug sensitivity testing plays an important role in the selection of more effective treatment regimens. Notably, the last organoid drug screening result showed highly sensitive to the treatment regimens with Sacituzumab govitecan, but this finding was unable to predict the overall clinical response of the patient, such as PR in the lung while PD in the CNS. This discordant response may be primarily attributed to the limited blood-brain barrier (BBB) penetration of most therapeutic agents and the inherent limitations of PDTOs in predicting intracranial response.

First, BBB is a highly selective physiological barrier composed of brain microvascular endothelial cells, tight junctions, astrocytes, and pericytes, which restricts the entry of most therapeutic agents into the CNS while maintaining brain homeostasis (24). In our study, the patient achieved PR in the lung lesion but failed to control CNS progression following treatment with organoid-guided Sacituzumab govitecan and Pembrolizumab. Sacituzumab govitecan, an antibody-drug conjugate composed of a humanized anti-Trop-2 antibody linked to SN-38, has shown potent extracranial antitumor activity in advanced solid tumors (25). Although it provides a promising therapeutic option for patients with brain metastases, particularly those with triple-negative breast cancer, its ability to penetrate the BBB remains limited (26). Similarly, Pembrolizumab, a programmed death-1 inhibitor, exhibits poor BBB penetration due to its large molecular weight (approximately 150 kDa), leading to low concentrations in the cerebrospinal fluid (CSF). Notably, however, such low CSF concentrations have been shown to be sufficient to functionally block PD-1 on T cells in the CNS (27). This distinction is critical, as the efficacy of immune checkpoint inhibitors such as Pembrolizumab against brain metastases depends more on the trafficking of peripherally activated T cells into the CNS than on the direct penetration of the drugs themselves across the BBB (28). Second, the PDTOs in our study were established from malignant pleural effusions, which could reflect the molecular and phenotypic characteristics of extracranial tumor cells but failed to recapitulate the unique microenvironment and biological features of intracranial tumors. PDTOs are cultured *in vitro* in the absence of the BBB and do not recapitulate interactions between tumor cells and brain-resident cells, such as astrocytes and microglia. Although glioblastoma organoids established in previous studies could retain some vascular endothelial cells, no mature and functional blood vessels were formed (29). Furthermore, intracranial tumor cells may undergo unique genetic and epigenetic alterations during metastasis, leading to differences in drug sensitivity compared to extracranial tumor cells (30). Therefore, PDTOs derived solely from extracranial lesions are insufficient to predict therapeutic responses in intracranial metastases. Future studies should focus on developing novel strategies to improve BBB penetration of therapeutic agents and establishing PDTOs from intracranial samples, such as CSF-derived tumor cells, to better predict intracranial drug response.

In conclusion, the patient with advanced lung adenocarcinoma of EGFR exon 21 L858R mutations achieved PR in the lung lesion following personalized treatment guided by LCOs established from patient-derived malignant pleural effusions, even after multiple lines of prior therapy. Our case demonstrates that LCOs may serve as a powerful platform for tailoring individualized treatment regimens for patients with advanced lung adenocarcinoma through rapid drug screening, and this personalized approach exhibits great potential of improving clinical outcomes.

## Data Availability

The original contributions presented in the study are included in the article/supplementary material. Further inquiries can be directed to the corresponding author.
